# Methodological Quality and Health Equity of AI Prediction Models in Rectal Cancer

**DOI:** 10.1111/jebm.70186

**Published:** 2026-08-18

**Authors:** Chengqian Ye, Zhaochu Wang, Qingjie Wu, Guosheng Lin, Cai Zhang

**Affiliations:** ^1^ Department of Gastroenterology The Affiliated People's Hospital of Fujian University of Traditional Chinese Medicine Fuzhou China; ^2^ Department of Anorectal Surgery The Affiliated People's Hospital of Fujian University of Traditional Chinese Medicine Fuzhou China; ^3^ Research Base of Traditional Chinese Medicine Syndrome Fujian University of Traditional Chinese Medicine Fuzhou China; ^4^ Department of Traditional Chinese Medicine The First Affiliated Hospital of Fujian Medical University Fuzhou China; ^5^ Department of Health and Kinesiology University of Illinois Urbana‐Champaign Urbana USA; ^6^ Beckman Institute for Advanced Science and Technology, University of Illinois Urbana‐Champaign Urbana USA

1

Rectal cancer care has become a sequence of high‐consequence, often irreversible decisions, and artificial intelligence (AI) prediction models have now been proposed as aids at nearly every decision point. Whether a patient is spared total mesorectal excision and routed to watch‐and‐wait organ preservation turns on predicting pathological complete response (pCR); whether occult nodal disease or extramural venous invasion (EMVI) is present, whether the tumor will recur, and whether surgery will leave a permanent stoma or disabling bowel dysfunction are each distinct prognostic problems with their own treatment and quality‐of‐life stakes. Across these tasks, radiomics, classical machine learning, and deep learning have all been advanced, and internally derived areas under the curve (AUC) routinely sit between 0.80 and 0.92. We conducted a structured, task‐stratified appraisal of a documented sample of 41 primary rectal cancer prediction model studies and 10 task‐level systematic reviews. Each was read against the Prediction model Risk Of Bias ASsessment Tool (PROBAST) [1], the Transparent Reporting of a multivariable prediction model for Individual Prognosis Or Diagnosis: AI extension (TRIPOD+AI) [2], and the PROBAST+AI extension [3]. The sampling frame, the model‐class taxonomy, and the pre‐specified exclusion of large‐language‐model applications are set out in Text S1. We reach one central conclusion: AI models for rectal cancer are discrimination‐rich but trustworthiness‐poor.

The evidence sorts into a maturity gradient defined not by headline discrimination but by the density of credible external evidence behind each task (Table 1). The pCR and nodal staging are the most developed, each anchored by multiple systematic reviews over dozens of primary studies, with nodal AI reproducibly outperforming the human reader (pooled radiomics AUC near 0.81 vs. radiologist near 0.66) [4, 5]. EMVI and survival occupy an intermediate band: modeling has advanced to T2‐weighted radiomic nomograms and to multitask deep learning fused with clinicopathologic data, and pooled or internal numbers look strong (EMVI pooled AUC near 0.91; recurrence AUC 0.80 to 0.97), yet credible generalizability narrows to a handful of externally validated exemplars [6, 7]. Surgical outcomes such as anastomotic leakage and permanent stoma are not immature so much as methodologically contested, and treatment toxicity is the thinnest task by a wide margin, with essentially no externally validated, calibration‐reported acute‐toxicity model and only a single modest prospective effort reporting a test area under the receiver operating characteristic curve (AUROC) of 0.71 [8]. No task has yet crossed from promising internal performance to demonstrated, externally validated readiness for routine clinical use.

**TABLE 1:** Task‐level maturity of AI prediction models in rectal cancer.

Clinical task	Evidence base	Dominant modality	Indicative AUC (internal vs. external validation)	Dominant PROBAST high‐risk domain	Geographic footprint
pCR after neoadjuvant therapy	Most developed; 2–3 anchor SR/MAs over dozens of primaries	MRI radiomics; DL on T2WI/DWI; radiopathomics	0.80–0.90 internal; 0.68–0.75 accuracy external	Analysis (low EPV, internal‐only, no calibration)	Predominantly Chinese/East Asian
Extramural venous invasion	about 8 primaries + 1 EMVI SR/MA	T2WI radiomic nomograms; one automated DL pipeline	0.74–0.96 internal; pooled 0.91; 0.78–0.83 external	Analysis (small sample size, vendor‐specific sequences)	Almost entirely single‐center Chinese
Lymph‐node/nodal staging	Dense; 2 anchor SR/MAs (8,039 patients)	Multiparametric MRI radiomics and DL nomograms	Pooled 0.81–0.92 internal; 0.78–0.82 external; peaks to 0.99	Analysis (overfitting/leakage, internal optimism)	Almost exclusively Chinese tertiary centers
Survival, recurrence, prognosis	Intermediate; multiple multitask‐DL primaries	Multiparametric MRI + clinicopathologic fusion	Recurrence AUC 0.80–0.97; C‐index falls 0.92 to 0.81	Analysis (fixed‐horizon binarization, sparse calibration)	Predominantly Chinese
Treatment toxicity	Thinnest; no validated acute‐toxicity model	Dose‐volume/AI‐segmented OAR metrics; clinical ML	Acute diarrhoea AUROC 0.71; late major‐LARS 0.835 to 0.884	Analysis (low event rate); outcome (surrogate endpoint)	Chinese LARS surrogates; UK multicenter trial (acute)
Surgical outcomes (AL, stoma)	Two 2025 anchor SRs + large primaries	Tabular clinical ML (XGBoost, RF, LASSO‐LR); post hoc SHAP	AL 0.63 nationwide to 0.984, falling to 0.703; stoma 0.612–0.987, external 0.82–0.89	Analysis (low event fraction; ML matching/under LR)	Nationwide Japan (AL); Chinese (stoma)

*Note*: Maturity reflects validation depth, calibration reporting, and generalizability, not AUC alone; counts and AUC ranges are indicative.

Abbreviations: AI, artificial intelligence; AL, anastomotic leakage; AUC, area under the curve; AUROC, area under the receiver operating characteristic curve; DL, deep learning; DWI, diffusion‐weighted imaging; EMVI, extramural venous invasion; EPV, events per variable; LARS, low anterior resection syndrome; LASSO, least absolute shrinkage and selection operator; LR, logistic regression; ML, machine learning; MRI, magnetic resonance imaging; OAR, organs at risk; pCR, pathological complete response; PROBAST, Prediction model Risk Of Bias ASsessment Tool; RF, random forest; SHAP, SHapley Additive exPlanations; SR/MA, systematic review/meta‐analysis; T2WI, T2‐weighted imaging; XGBoost, extreme gradient boosting.

Beneath the gradient, the same structural weaknesses recur regardless of task or modality (Table S1), signaling a field‐wide methods problem rather than isolated study flaws, although the mechanism by which each weakness arises is class‐specific (Table S2). Reported discrimination is almost always an internal, optimism‐prone estimate produced under single‐center retrospective design, low events‐per‐variable, complete‐case handling of missing data, unspecified decision thresholds, and unreported calibration, precisely the analysis‐domain failures that PROBAST and PROBAST+AI are built to surface. This matches cross‐specialty base rates, where roughly 87% of machine‐learning prediction studies are rated high risk of bias, overwhelmingly on the analysis domain [9]. Risk of bias and validation status behave as effect modifiers on pooled performance rather than as incidental descriptors: in the largest pCR synthesis, pooled estimates were significantly higher in internally than externally validated models, in single‐center than multi‐center designs, and in Asian than non‐Asian populations [5]. Internal‐to‐external decay is the modal behavior of the field, from a survival concordance index falling from 0.92 to 0.81 [7] to, at the extreme, an anastomotic‐leakage AUC collapsing from 0.984 to 0.703 [10]. The fair comparison that would justify model complexity is rarely made: of the 30 studies in our sample whose primary model was a flexible learner, only 7 reported a regression model fitted on the same cohort, so in 77% the added complexity was never benchmarked against the obvious alternative (Table S3). Where it is made, flexible machine learning frequently fails to earn that complexity: in a nationwide analysis of 119,818 cases, XGBoost reached an AUROC of 0.633 against logistic regression at 0.632, with no significant difference [11], and the anchor anastomotic‐leakage review found machine learning trained on smaller cohorts, carrying higher risk of bias, and scoring no better on TRIPOD+AI transparency than regression [12]. Implausibly high single‐center AUCs approaching 0.99 in nodal and stoma models are most parsimoniously read as overfitting or leakage rather than transportable performance, and the modal mechanisms are specific: partitioning at the image or lesion level rather than the patient level, so that slices from one patient fall on both sides of the split, and performing feature selection or scanner harmonization on the full dataset before that split. No study in our full‐text subset stated explicitly that partitioning was done at the patient level (Table S3).

These failures rest on narrow data foundations, the single most consequential threat to transportability. Models are developed on retrospective cohorts from one or a few academic centers, scanned on a handful of platforms, and drawn from demographically homogeneous populations; 38 of the 41 studies we appraised used development data from a single country (Table S3). Handcrafted radiomic features, which still dominate the imaging tasks, shift systematically across scanners and vendors: under intensity normalization alone only 9 to 10 of 93 features remained comparable across institutions, whereas ComBat feature‐distribution harmonization restored 79 of 93 [13]. Unharmonized discrimination is therefore better understood as a scanner‐specific than a disease‐specific quantity. Geographic concentration compounds the problem, because even the field's strongest external validations are typically repeated within a single, mostly Chinese, healthcare system, so transport across countries, systems, and patient ancestries is essentially untested. The equity consequence is starker still: none of the 41 models reported demographic representativeness, race or ethnicity subgroup performance, or an algorithmic fairness audit, and a dedicated search on July 28, 2026 (Text S1) identified no rectal cancer exception. The nearest adjacent evidence is instructive rather than reassuring, since the one colorectal study that did test fairness found performance disparities by age, sex, and race in every one of the five classifiers it examined (Text S1). The mechanism is data absenteeism, whereby groups missing from the source data become invisible to the model and to any post hoc audit, so fairness cannot be recovered once the underlying records were never collected [14].

The corrective agenda follows directly and is operable today. First, flexible machine learning should not be deployed until it demonstrably beats a well‐specified logistic‐regression baseline on a shared, adequately powered cohort, the two fitted and reported side by side. Second, geographic, ideally cross‐country and prospective, external validation should move from rare exception to entry requirement, since the 0.10 to 0.15 (and occasionally near 0.28) internal‐to‐external decay defines the gap between promise and transportability [10]. Two mechanisms make this tractable without moving patient records across borders, and both are already demonstrated in this disease: cross‐institutional federated learning, in which the model travels to the data, and shared international benchmark repositories with sequestered test sets (Text S1). Third, calibration should be reported where the decision is actually made rather than only in aggregate. Our full‐text subset shows the field performing the form of clinical‐utility assessment without its substance: decision‐curve analysis appeared in 62% of studies and calibration curves in 54%, yet calibration slope or intercept appeared in 1 of 24 and a clinically motivated threshold in 2 of 24, while the Youden index, which weights a false positive and a false negative equally, was three times more common (Table S3). Nothing about organ preservation versus a permanent stoma is symmetric, so calibration intercept and slope within threshold‐relevant risk strata, and net benefit at the pre‐specified threshold, should accompany every discrimination metric. Fourth, feature‐distribution harmonization and automated, leakage‐resistant segmentation should be treated as prerequisites for any imaging model meant to work outside its training scanner [13]. Fifth, representativeness and subgroup fairness should be engineered as pre‐registered design inputs rather than retrospective checks. Sixth, interpretability should be designed in rather than appended as a post hoc SHapley Additive exPlanations (SHAP) plot that explains an unvalidated model, and conformance to TRIPOD+AI [2] and PROBAST+AI [3] should become a condition of publication. The field's rare positive exemplars show this is achievable: a permanent‐stoma model that held an external AUC of 0.815 and shipped as a public, auditable calculator is the default the field should adopt, and it was the only publicly deployed tool among the 41 [15].

What sets rectal cancer AI apart is the combination of unusually high‐stakes, often irreversible decisions layered on weak, geographically narrow data foundations with fairness evidence that a dedicated search confirms is absent rather than merely sparse. This warrants a validation‐first rather than performance‐first stance. Converting promising AUCs into deployable, equitable tools will depend less on further internal‐only model proliferation and more on externally validated, calibrated, transparent, and equity‐aware development under standards such as TRIPOD+AI, PROBAST+AI, and the reporting guideline for the Developmental and Exploratory Clinical Investigations of Decision support systems driven by AI (DECIDE‐AI), conformance to which is now among the rate‐limiting steps in the clinical translation of AI for rectal cancer.

## Conflicts of Interest

All authors declare no conflicts of interest.

## Supporting information




**Text S1**: Supplementary methods.
**Table S1**: Recurring methodological pitfalls and the reporting standardsthat addressthem.
**Table S2**: Model class‐specific failure modes, leakage mechanisms, and the signaling questionsthat detectthem.
**Table S3**: Quantified appraisal ofthe 41‐study sample.
