# Supplementary material for: Methodological Quality and Health Equity of AI Prediction Models in Rectal Cancer
Source: J Evid Based Med. 2026 Aug 18;19(3):e70186. doi: 10.1111/jebm.70186 (PMC13613241; doi:10.1111/jebm.70186)
Supplement: Supplementary file 1 [file JEBM-19-0-s001.docx]

**Supplemental Materials**

**Methodological quality and health equity of AI prediction models in rectal cancer**

[Text S1 Supplementary Methods	2](#_Toc236693966)

[1. Design and scope	2](#_Toc236693967)

[2. Sampling frame and search	2](#_Toc236693968)

[3. Selection, inclusion, and sensitivity check	3](#_Toc236693969)

[4. Model classes	3](#_Toc236693970)

[5. Appraisal instruments	4](#_Toc236693971)

[6. Scope exclusions	4](#_Toc236693972)

[7. Equity verification	4](#_Toc236693973)

[8. Coding, ascertainment, and limitations	5](#_Toc236693974)

[Table S1 Recurring methodological pitfalls and the reporting standards that address them	8](#_Toc236693975)

[Table S2 Model-class-specific failure modes, leakage mechanisms, and the signalling questions that detect them	9](#_Toc236693976)

[Table S3 Quantified appraisal of the 41-study sample	10](#_Toc236693977)

[REFERENCES	11](#_Toc236693978)

Text S1 Supplementary Methods

1. Design and scope

This Letter reports a structured, task-stratified meta-research appraisal of artificial-intelligence (AI) prediction models across the major clinical tasks of rectal-cancer care: pathological complete response after neoadjuvant therapy, extramural venous invasion, lymph-node metastasis and nodal staging, survival and recurrence, treatment toxicity, and surgical outcomes (anastomotic leakage and permanent stoma). The unit of appraisal is the study rather than the algorithm, the appraisal instrument was fixed before extraction, and the design follows an unregistered internal protocol (version 1.0, dated 25 June 2026). It is not a systematic review and not a meta-analysis: we did not dual-independently screen a complete multi-database yield, we present no PRISMA flow diagram, and no effect estimate is pooled. Every pooled figure quoted in the Letter is taken from, and attributed to, a published task-level synthesis.

2. Sampling frame and search

The frame was built from a PubMed search executed on 25 June 2026 and refreshed on 28 July 2026, combining three concept blocks with AND: (i) a recall-first rectal and colorectal population block ("Rectal Neoplasms"[Mesh] OR "Colorectal Neoplasms"[Mesh] OR rectal[tiab] OR rectum[tiab] OR colorectal[tiab] OR LARC[tiab] OR "anastomotic leak*"[tiab] OR "anterior resection"[tiab] OR stoma[tiab]); (ii) an AI block ("Artificial Intelligence"[Mesh] OR "Machine Learning"[Mesh] OR "Deep Learning"[Mesh] OR radiomic*[tiab] OR "neural network*"[tiab] OR "prediction model*"[tiab] OR nomogram*[tiab]); and (iii) a prediction-context block (predict* OR prognos* OR diagnos* OR staging OR response OR recurrence OR survival OR outcome* OR risk, all [tiab]). A secondary-study layer ("systematic review"[pt] OR "meta-analysis"[pt] OR the corresponding text words) was applied on top for the review stream.

The date floor was 2015, because the first rectal-cancer radiomics and machine-learning prediction studies appear from that year, and no language limit was applied. Yields were 10,086 records for the primary stream, 1,170 for the review stream, and 1,058 for the review stream since 2015.

3. Selection, inclusion, and sensitivity check

Task-level systematic reviews were selected as anchors, taking the largest and most recent review for each task where one existed. Primary studies were harvested from those anchors and, for tasks with no dedicated review (extramural venous invasion, treatment toxicity), by forward and backward citation chasing from them. Inclusion required a rectal-cancer population or a separable rectal subgroup, an AI, machine-learning or radiomics prediction model, and at least one reported discrimination metric.

Sensitivity of the frame was checked against a pre-specified seed set of nine known single-task reviews[1-9], all nine of which the search string of record captured. The resulting set is 41 primary prediction-model studies and 10 task-level systematic reviews or meta-analyses. Five candidate records were excluded at this step for population (metastatic colorectal liver metastases; anal squamous cell carcinoma; colon anastomosis) or article type (one systematic review reclassified to the anchor group, one editorial).

4. Model classes

Studies were assigned to one of four model classes: handcrafted radiomics, deep learning on images, tabular clinical machine learning, and fused radiopathomics or multimodal models. Class is recorded for every study in Table S3, and class-specific failure modes are set out in Table S2. Classes were appraised on a common scaffold because the domains of the Prediction model Risk Of Bias ASsessment Tool (PROBAST) and the items of the Transparent Reporting of a multivariable prediction model for Individual Prognosis Or Diagnosis: Artificial Intelligence extension (TRIPOD+AI) are defined over the design and analysis of a prediction-model study rather than over the estimator used.

5. Appraisal instruments

Each task and study was read against PROBAST, which evaluates the participants, predictors, outcome and analysis domains[10,11]; the TRIPOD+AI reporting standard, a 27-item floor for transparent development and validation of machine-learning prediction models[12]; and the PROBAST+AI extension, which targets AI-specific failure modes including data leakage, optimistic resampling, and inadequate handling of model complexity[13,14]. DECIDE-AI was invoked where a task approached the clinical-deployment stage.

6. Scope exclusions

Large-language-model applications were excluded before extraction. A PubMed check on 28 July 2026 returned 196 records for colorectal cancer combined with large language models, GPT or ChatGPT; that literature, as characterized by its own systematic review of 37 studies[9], is confined to knowledge assessment, question answering, and clinical-documentation support. We identified no large-language-model rectal-cancer risk-prediction model reporting discrimination and calibration.

7. Equity verification

The Letter states that no rectal-cancer model reports demographic representativeness, race or ethnicity subgroup performance, or an algorithmic fairness audit. That statement was verified in two ways rather than inferred from the sampled studies alone. First, fairness and subgroup reporting was coded as an explicit item for all 41 studies, at full text for the 24 with open-access full text and at abstract level for the remainder; the count was zero in both strata.

Second, a dedicated search was run on 28 July 2026. A broad string combining the rectal and colorectal population and AI blocks with (fairness OR "algorithmic bias" OR equity OR equitable OR disparit* OR "subgroup performance" OR race OR ethnicity OR representativeness) returned 251 records, and a strict string restricted to fairness, algorithmic fairness and algorithmic bias returned 10 records, which were screened individually. Nine unique studies remained after deduplication, and none was a fairness or subgroup-performance audit of a rectal-cancer prediction model: five were narrative reviews naming algorithmic bias as a risk without any model-level subgroup analysis, one was a survey of screening professionals, one was a keyword artifact (a lung-cancer screening study drawing on the Prostate, Lung, Colorectal and Ovarian trial), and two were data-infrastructure papers addressing fairness at the repository rather than the model level[15,16].

The nearest adjacent evidence is a study[17] that compared five machine-learning classifiers of multicategory survival outcome in colorectal cancer using The Cancer Genome Atlas and found performance disparities across age, sex and racial groups in all five. That study is colorectal rather than rectal, and its task does not correspond to any of the six tasks appraised here.

8. Coding, ascertainment, and limitations

Items were coded by one reviewer against a fixed form and then independently checked against the source record by a second reviewer, with disagreements resolved by discussion; the study-level data are reported in full in Table S3 so that any reader can recount them. External validation, geographic footprint, model class, regression comparator and public availability were ascertainable for all 41 studies. Calibration statistics, threshold derivation, and partitioning detail require full text and were coded on the PubMed Central open-access subset, for which full text was retrievable for 24 of 41 (59%); those rows carry the smaller denominator explicitly and are never pooled with abstract-level rows.

Two limitations bound the interpretation. First, this is a purposive documented sample rather than a complete screened yield, and it was assembled to include the field’s strongest exemplars, so it is enriched for externally validated work relative to the underlying literature; the proportions in Table S3 are therefore conservative as a critique, and field-wide rates are better estimated from the anchor syntheses. Second, although every item was checked by a second reviewer, verification against a completed form is not equivalent to dual independent extraction, and no formal agreement statistic was computed; independent duplicate extraction remains the appropriate next step and lies beyond the scope of a Research Letter.

Table S1 Recurring methodological pitfalls and the reporting standards that address them

| **Recurring pitfall** | **Where it recurs** | **Standard / reporting item that addresses it** |
| --- | --- | --- |
| No external validation (internal-only optimism) | pCR, EMVI, nodal, survival, AL, stoma; 19 of 41 studies had none | PROBAST Analysis (overfitting/evaluation); TRIPOD+AI validation-data-source items; PROBAST+AI validation signaling questions. Recommend geographic, ideally cross-country and prospective, external validation as a gate. Where data cannot cross borders, cross-institutional federated learning[18-20] and shared benchmark repositories with sequestered test sets[15,16] make the gate achievable. |
| Single-center/single-source cohort | All tasks; 38 of 41 studies used single-country development data | PROBAST Participants (appropriate data source); TRIPOD+AI participants/data-provenance and fairness items documenting representativeness. |
| Low events-per-variable/small samples | EMVI, survival nomograms, toxicity, AL/stoma | PROBAST Analysis (adequate outcome events/EPV); TRIPOD+AI sample-size-justification item. |
| No pre-specified probability threshold | nodal staging, AL, stoma; a clinically motivated threshold appeared in 2 of 24 full texts, the Youden index in 6 | PROBAST Analysis (a priori thresholds); TRIPOD+AI risk-categorization and threshold items. The threshold must be tied to the decision: the low-risk region that would route a patient to watch-and-wait organ preservation, and the high-risk region that would trigger a defunctioning or permanent stoma. The Youden index weights false positives and false negatives equally and is therefore the wrong rule for both. |
| No calibration reporting, or calibration reported only in aggregate | pCR, EMVI, survival, most tasks; calibration slope or intercept appeared in 1 of 24 full texts | PROBAST Analysis (calibration/overfitting); TRIPOD+AI calibration item. Report calibration intercept and slope within threshold-relevant risk strata, not a single curve across the whole range, and pair with net benefit evaluated at the pre-specified threshold. |
| No fairness/subgroup audit | All tasks; 0 of 41 studies, and no exception found on dedicated search (Text S1 §7) | TRIPOD+AI fairness items; PROBAST+AI applicability; DECIDE-AI for equitable deployment. |
| Data leakage/implausibly high internal AUC | nodal (0.99), stoma (0.987), small-sample EMVI; 0 of 24 full texts stated patient-level partitioning explicitly | PROBAST+AI development signaling questions on partitioning/leakage; PROBAST Analysis (optimism); TRIPOD+AI train/test-separation items. Modal mechanisms are class-specific (Table S2). |
| Censored survival collapsed to fixed-horizon binary | survival, recurrence, metastasis, late-toxicity surrogates | PROBAST Outcome (appropriate definition); TRIPOD+AI outcome item; use time-to-event modelling with Harrell concordance, KM stratification, calibration-in-time. |
| Post hoc interpretability bolted on (SHAP after the fact) | AL, stoma, EMVI | TRIPOD+AI model-specification and explainability items; PROBAST+AI model-quality questions; design interpretability in. |
| Flexible ML without a regression baseline | AL, stoma, nodal staging; 23 of 30 flexible-learner studies reported none | TRIPOD+AI model-comparison items; parallel logistic-regression and machine-learning development on shared, adequately powered cohorts. |
| Vendor/scanner-specific or non-harmonized features | pCR, EMVI, nodal; generalizability broadly | TRIPOD+AI data-provenance and preprocessing items; apply ComBat harmonization; PROBAST Predictors (reproducible assessment). |
| No prospective registration/no code or model availability | most tasks; 1 of 41 studies shipped a public calculator | TRIPOD+AI protocol/registration and model/code/data-availability items; DECIDE-AI reporting for live clinical evaluation. |

AL, anastomotic leakage; AUC, area under the curve; DECIDE-AI, reporting guideline for the Developmental and Exploratory Clinical Investigations of DEcision support systems driven by Artificial Intelligence; EMVI, extramural venous invasion; EPV, events per variable; KM, Kaplan-Meier; LR, logistic regression; ML, machine learning; pCR, pathological complete response; PROBAST, Prediction model Risk Of Bias ASsessment Tool; PROBAST+AI, PROBAST Artificial Intelligence extension; SHAP, SHapley Additive exPlanations; TRIPOD+AI, Transparent Reporting of a multivariable prediction model for Individual Prognosis Or Diagnosis: Artificial Intelligence extension. Mapped to PROBAST, TRIPOD+AI, PROBAST+AI, and DECIDE-AI. Counts refer to the appraisal set described in Text S1 and coded in Table S3.

Table S2 Model-class-specific failure modes, leakage mechanisms, and the signaling questions that detect them

| **Model class** | **Modal leakage mechanism** | **Modal analysis-domain failure** | **Class-specific transportability threat** | **Detecting item** |
| --- | --- | --- | --- | --- |
| Handcrafted radiomics (with LASSO or regression nomogram) | Feature selection, ComBat harmonization, or normalization fitted on the full dataset before the train/test split, so test-set information enters feature construction | Low events-per-variable relative to feature count; optimism uncorrected because resampling reuses the selected feature set | Feature values shift systematically across scanners and vendors; under intensity normalization alone only 9 to 10 of 93 features remained comparable across institutions[21] | PROBAST+AI partitioning and preprocessing signaling questions; TRIPOD+AI preprocessing and data-provenance items |
| Deep learning on images | Partitioning at the image, slice or lesion level rather than the patient level, so images from one patient appear on both sides of the split; outcome-aware manual segmentation by an unblinded reader | Optimism from architecture and hyperparameter selection on the same held-out data used to report performance | Learned features encode acquisition protocol and scanner as much as tumor biology; sequence availability (DCE, DKI, synthetic MRI) is not portable | PROBAST+AI development signaling questions on data partitioning and model complexity; TRIPOD+AI train/test-separation item |
| Tabular clinical machine learning | Target leakage from post-operative or post-treatment variables into a model presented as pre-operative; threshold and hyperparameter selection on the test set; repeated resampling without nested cross-validation | Very low event fraction (anastomotic leakage, permanent stoma); complete-case handling of missing peri-operative data | Case mix, referral patterns and coding practice differ across health systems more than imaging does; nationwide performance is far below single-center reports (AUROC 0.63 versus 0.98) | PROBAST Analysis (EPV, missing data, optimism); TRIPOD+AI model-comparison item requiring a regression baseline |
| Fused radiopathomics and multimodal models | Modality-specific preprocessing pipelines fitted before the split; slide-level and patient-level units conflated when whole-slide images are tiled | Effective sample size set by the scarcest modality while the parameter count is set by the richest | Requires simultaneous availability of MRI, whole-slide pathology and structured clinical data, which few centers outside the development site can supply | PROBAST+AI model-quality and applicability questions; TRIPOD+AI data-provenance and model-specification items |

AI, artificial intelligence; AUROC, area under the receiver operating characteristic curve; DCE, dynamic contrast-enhanced; DKI, diffusion kurtosis imaging; EPV, events per variable; LASSO, least absolute shrinkage and selection operator; MRI, magnetic resonance imaging; PROBAST, Prediction model Risk Of Bias ASsessment Tool; PROBAST+AI, PROBAST Artificial Intelligence extension; TRIPOD+AI, Transparent Reporting of a multivariable prediction model for Individual Prognosis Or Diagnosis: Artificial Intelligence extension. The Letter’s claim is that the analysis-domain failures recur across classes while the mechanisms that generate them do not. This table is the basis for that distinction and for the leakage mechanisms named in the main text. No study in the open-access full-text subset stated explicitly that partitioning was performed at the patient level (Table S3), which is the single reporting item that would most cheaply exclude the deep-learning mechanism above.

Table S3 Quantified appraisal of the 41-study sample

| **Item** | **k/N** | **%** | **Denominator and ascertainment** |
| --- | --- | --- | --- |
| Any external validation | 22/41 | 54 | All studies; abstract and results text |
| External validation in a different country | ≤1/41 | ≤2 | All studies; only one study[22] (9 European centres) is a candidate |
| Development data from a single country | 38/41 | 93 | All studies |
| Development data from China or Other places | 33/41 | 80 | All studies |
| Publicly available model, code, or calculator | 1/41 | 2 | All studies; permanent-stoma calculator[23] |
| Any fairness or subgroup-performance audit | 0/41 | 0 | All studies; full text for 24, abstract for 17; corroborated by the dedicated search in Text S1 §7 |
| Flexible learner reported alongside a same-cohort regression model | 7/30 | 23 | Restricted to the 30 studies whose primary model was a flexible learner; in radiomics nomogram studies logistic regression is the model, not a comparator |
| Flexible learner with no regression comparator | 23/30 | 77 | As above |
| Decision-curve or net-benefit analysis | 15/24 | 62 | PubMed Central open-access full-text subset |
| Calibration curve or plot shown | 13/24 | 54 | Open-access full-text subset |
| Brier score reported | 4/24 | 17 | Open-access full-text subset |
| Hosmer-Lemeshow as the calibration statistic | 4/24 | 17 | Open-access full-text subset |
| Calibration slope or intercept reported | 1/24 | 4 | Open-access full-text subset |
| Threshold set by the Youden index (data-derived) | 6/24 | 25 | Open-access full-text subset |
| Clinically motivated decision threshold | 2/24 | 8 | Open-access full-text subset |
| Explicit statement of patient-level data partitioning | 0/24 | 0 | Open-access full-text subset |

k, number of studies meeting the item; N, denominator for that item. Study-level coding is available in full from the authors. Rows with denominator 41 were ascertainable from the abstract and indexed results text for every study; rows with denominator 24 required full text and were coded on the PubMed Central open-access subset (24 of 41, 59%). The two denominators are reported separately and are never pooled. The sample is purposive and was assembled to include the field’s strongest exemplars, so it is enriched for externally validated work relative to the underlying literature; these proportions are therefore conservative as a critique (Text S1 §8). The juxtaposition of rows 9 to 16 is the substantive finding: clinical-utility assessment is performed in form, with decision curves in 62% and calibration curves in 54%, but not in substance, with calibration slope or intercept in 1 of 24 and a clinically motivated threshold in 2 of 24.

REFERENCES

1. Jong BK, Yu ZH, Hsu YJ, Chiang SF, You JF, Chern YJ. Deep learning algorithms for predicting pathological complete response in MRI of rectal cancer patients undergoing neoadjuvant chemoradiotherapy: a systematic review. *International Journal of Colorectal Disease.* 2025;40(1):19.

2. Shen H, Jin Z, Chen Q, Zhang L, You J, Zhang S, et al. Image-based artificial intelligence for the prediction of pathological complete response to neoadjuvant chemoradiotherapy in patients with rectal cancer: a systematic review and meta-analysis. *La Radiologia Medica*. 2024;129(4):598-614.

3. Boldrini L, Charles-Davies D, Romano A, Mancino M, Nacci I, Tran HE, et al. Response prediction for neoadjuvant treatment in locally advanced rectal cancer patients: improvement in decision-making: a systematic review. *European Journal of Surgical Oncology*. 2024;51(7):109463.

4. Abbaspour E, Karimzadhagh S, Monsef A, Joukar F, Mansour-Ghanaei F, Hassanipour S. Application of radiomics for preoperative prediction of lymph node metastasis in colorectal cancer: a systematic review and meta-analysis. *International Journal of Surgery*. 2024;110(6):3795-3813.

5. Ben Hmido S, Abder Rahim H, Ingwersen EW, Burchell G, Hompes R, van der Peet D, et al. Transparency in externally validated models: a systematic review of machine learning vs. logistic regression for predicting colorectal anastomotic leakage. *European Journal of Surgical Oncology*. 2025;51(10):110367.

6. Tan W, Cai S, Wu J, Wu W, Wang S, Li Y, et al. Risk prediction models for permanence of temporary stoma after radical surgery of rectal cancer: a systematic review. *World Journal of Surgical Oncology*. 2025;23(1):246.

7. Chen K, Qu Y, Han Y, Li Y, Gao H, Zheng D. Performance of machine learning in diagnosing KRAS mutations in colorectal cancer: systematic review and meta-analysis. *Journal of Medical Internet Research*. 2025;27:e73528.

8. Yu S, Gong M, Wang H, Liu H, Deng M. Radiomics and artificial intelligence-based prediction of tumor response in digestive system neoplasm: a systematic review and meta-analysis. *Frontiers in Medicine*. 2026;13:1795060.

9. Tian J, Lou Q, Wang X, Xu H, Mei H, Yu Y. Large language models in colorectal cancer care and clinical decision support: systematic review. *Journal of Medical Internet Research*. 2026;28:e89862.

10. Wolff RF, Moons KGM, Riley RD, Whiting PF, Westwood M, Collins GS, et al. PROBAST: a tool to assess the risk of bias and applicability of prediction model studies. *Annals of Internal Medicine*. 2019;170(1):51-58.

11. Moons KGM, Wolff RF, Riley RD, Whiting PF, Westwood M, Collins GS, et al. PROBAST: a tool to assess risk of bias and applicability of prediction model studies: explanation and elaboration. *Annals of Internal Medicine*. 2019;170(1):W1-W33.

12. Collins GS, Moons KGM, Dhiman P, Riley RD, Beam AL, Van Calster B, et al. TRIPOD+AI statement: updated guidance for reporting clinical prediction models that use regression or machine learning methods. *BMJ*. 2024;385:e078378.

13. Collins GS, Dhiman P, Andaur Navarro CL, Ma J, Hooft L, Reitsma JB, et al. Protocol for development of a reporting guideline (TRIPOD-AI) and risk of bias tool (PROBAST-AI) for diagnostic and prognostic prediction model studies based on artificial intelligence. *BMJ Open*. 2021;11(7):e048008.

14. Yan MH, Hu LL, Hu B, Li W. Interpretation of PROBAST-AI: a tool for assessing the quality, risk of bias, and applicability of prediction models based on regression or artificial intelligence methods. *Chinese Journal of Epidemiology*. 2026;47(5):958-964.

15. Tsave O, Kosvyra A, Filos DT, Fotopoulos DT, Chouvarda I. A multi-dimensional framework for data quality assurance in cancer imaging repositories. *Cancers*. 2025;17(19):3213.

16. Aghakhanyan G, Barucci A, Pascali MA, Assante M, Bagnacci G, Bertelli E, et al. NAVIGATOR: a regional multimodal imaging biobank initiative powered by AI tools for precision medicine in oncology. *European Journal of Radiology*. 2025;191:112327.

17. Feng CH, Deng F, Disis ML, Gao N, Zhang L. Towards machine learning fairness in classifying multicategory causes of deaths in colorectal or lung cancer patients. *Briefings in Bioinformatics*. 2025;26(4):bbaf398.

18. Mateus P, Savino M, Capocchiano ND, Berbee M, Gambacorta MA, Chiloiro G, et al. Predicting near-complete pathological response to (chemo)radiotherapy in patients with rectal cancer: a federated learning study. *Medical Physics*. 2025;52(8):e18034.

19. Maurer MM, Pfitzner B, van de Water RP, Faraj L, Riepe C, Zuluaga D, et al. Privacy preserving federated learning for 90-day mortality prediction in colorectal surgery: a multicenter retrospective development and comparison study. *International Journal of Surgery*. 2025;111(12):9065-9074.

20. Babendererde N, Lemke N, Stieber J, Fuchs M, Weng Z, Eich ML, et al. Nationwide federated learning for histopathology: secure deployment across Germany behind firewalls. *npj Digital Medicine*. 2026;9(1):581.

21. Giannini V, Panic J, Regge D, Balestra G, Rosati S. Could normalization improve robustness of abdominal MRI radiomic features? *Biomedical Physics & Engineering Express*. 2023;9(5):055018.

22. Cai L, Lambregts DMJ, Beets GL, Maas M, Pooch EHP, Guerendel C, et al. An automated deep learning pipeline for EMVI classification and response prediction of rectal cancer using baseline MRI: a multi-centre study. *npj Precision Oncology*. 2024;8(1):17.

23. Su Y, Li Y, Zhang H, Yang W, Liu M, Luo X, et al. Machine learning model for prediction of permanent stoma after anterior resection of rectal cancer: a multicenter study. *European Journal of Surgical Oncology*. 2024;50(7):108386.
